# Bio-Inspired Metaheuristics for Time-Optimal Trajectory Planning in Cooperative Dual-Arm Bimanipulation

**DOI:** 10.3390/biomimetics11030173

**Published:** 2026-03-02

**Authors:** Mario Peñacoba-Yagüe, Jesús-Enrique Sierra-García, Matilde Santos-Peñas

**Affiliations:** 1 Department of Digitalization, University of Burgos, 09001 Burgos, Spain; 2 Institute of Knowledge Technology, University Complutense of Madrid, 28040 Madrid, Spain; msantos@ucm.es

**Keywords:** cooperative dual-arm manipulation, time-optimal trajectory planning, bio-inspired metaheuristics, collision avoidance, industrial robotics

## Abstract

This paper addresses the generation of time-efficient, collision-free cooperative motions for a dual-arm robotic system transporting a shared payload in constrained industrial workspaces. Trajectory generation is formulated as a constrained optimization problem and solved through bio-inspired metaheuristic search, where candidate solutions are evaluated with a safety-first cost function that first enforces feasibility by heavily penalizing collisions and then minimizes total execution time among collision-free trajectories. Particle Swarm Optimization (PSO), Whale Optimization Algorithm (WOA), and Gazelle Optimization Algorithm (GOA) are evaluated under identical bounds and stopping conditions, showing that all three reliably discover feasible cooperative trajectories; however, clear differences emerge in feasibility discovery and final trajectory quality: PSO reaches feasibility earlier and achieves the lowest final objective value and the shortest trajectory execution time (6.825 s), followed by WOA (7.330 s) and GOA (8.525 s). Overall, this work contributes an object-centric optimization methodology for constrained dual-arm bimanipulation using bio-inspired metaheuristics, a feasibility-first cost structuring that explicitly separates safe motion discovery from time-optimal refinement, and a controlled benchmarking of PSO/WOA/GOA that quantifies their distinct convergence and late-stage refinement behaviors.

## 1. Introduction

Cooperative robotic bimanipulation has become a key capability for modern industrial automation, particularly in handling operations where a single manipulator cannot guarantee stable grasping, safe transport, or controlled reorientation of the workpiece. Inspired by human two-handed manipulation, dual-arm systems enhance object stability, enable deliberate reorientation, and distribute loads and moments between manipulators—capabilities that are especially relevant when manipulating bulky, elongated, or heavy payloads. Such objects typically induce large gravitational moments and inertial effects that challenge unilateral manipulation, especially when motion must be executed through narrow passages or near surrounding equipment and obstacles [1]. These advantages, however, come at the cost of increased planning complexity, since the motion must remain feasible for two kinematic chains simultaneously while respecting workspace limits, avoiding collisions, and preserving cooperative constraints throughout the task [2].

Trajectory planning for dual-arm bimanipulation is intrinsically constrained and highly nonconvex. Even when the task is specified at a high level (e.g., moving an object from an initial pose to a target pose), the feasible space is shaped by robot kinematics, joint limits, potential singularities, and the presence of obstacles and self-collision configurations [3]. In practical cells, additional constraints arise from tight clearances and the requirement to maintain safe separation from the environment while executing coordinated motion. As a result, classical gradient-based optimization methods can struggle due to discontinuities in collision-related cost terms and the prevalence of local minima, while purely sampling-based planners may produce feasible trajectories that are not time-efficient, require extensive tuning, or become computationally expensive as constraints intensify [4].

At the same time, industrial deployment demands not only feasibility but also efficiency: cycle time remains a central performance indicator, and even small reductions in execution time can translate into meaningful productivity gains [5]. Achieving time-efficient motion in constraint-dominated environments is particularly challenging in cooperative manipulation, since both manipulators must remain synchronized and collision-free while transporting an object that may sweep a large, occupied volume through the workspace [6]. These requirements motivate planning approaches that can reliably find feasible cooperative trajectories under tight constraints and subsequently refine them toward shorter execution times without compromising safety.

Bio-inspired metaheuristics provide an attractive alternative for such problems because they operate without requiring differentiability and can explore complex search spaces through population-based mechanisms and stochastic operators [7]. Methods such as Particle Swarm Optimization (PSO), Whale Optimization Algorithm (WOA), and Gazelle Optimization Algorithm (GOA) have shown strong empirical performance in difficult optimization landscapes, particularly when constraints and discontinuities make classical approaches brittle [8]. Nevertheless, their effectiveness in dual-arm bimanipulation depends critically on how feasibility is enforced. If collision avoidance is treated as a soft objective that competes directly with time minimization, the search may spend large effort exploring infeasible regions or converge prematurely to trajectories that are nominally short but unsafe or physically invalid [9]. To address this, the present work adopts a feasibility-first evaluation strategy that prioritizes collision-free motion before time refinement.

This work addresses these challenges by formulating cooperative dual-arm trajectory generation as a constrained optimization problem solved via bio-inspired metaheuristic search, combined with a feasibility-first evaluation strategy. Candidate trajectories are assessed with a stepwise cost structure that prioritizes safety by heavily penalizing collisions and infeasibility, and only after feasibility is satisfied does the cost drive the optimization toward reducing total execution time. Within this framework, a comparative assessment of three representative metaheuristics—PSO, WOA, and GOA—is conducted under consistent bounds and stopping conditions, focusing on their ability to converge reliably to collision-free cooperative motions and then improve time performance among feasible solutions.

The main contributions of this paper can be summarized as follows:

1. An object-centric methodology for cooperative dual-arm bimanipulation trajectory planning, formulated as a constrained optimization problem.
2. A feasibility-first, stepwise cost function that explicitly separates constraint satisfaction from time reduction, enhancing robustness in discontinuous and constraint-dominated scenarios.
3. A controlled comparative evaluation of PSO, WOA, and GOA for the proposed problem, highlighting their convergence behavior and effectiveness in optimizing cooperative, collision-free trajectories.

The remainder of the paper is organized as follows. Section 2 describes the related works. Section 3 presents the optimization framework and the stepwise cost function and outlines the metaheuristic algorithms considered. Section 4 details the experimental setup and evaluation procedure. Section 5 discusses the results and comparative findings. Finally, Section 6 concludes the paper and outlines directions for future work, including extensions to broader task families and additional constraints relevant to real industrial deployments.

## 2. Related Works

Dual-arm (bimanual) manipulation has regained strong momentum in recent years, driven by the need to handle larger objects, perform contact-rich operations, and increase robustness through coordinated behaviors. A key enabler for structuring this problem is the emergence of modern representations and benchmarks that make bimanual coordination more systematic. Krebs and Asfour proposed a bimanual manipulation taxonomy that organizes coordination patterns by coupling, symmetry and hand roles, providing a practical lens to formalize bimanual behaviors and derive constraints for robotic execution [10]. In parallel, learning-centric bimanual research has been boosted by benchmark environments and coordination-oriented policy designs. Chen et al. extends reinforcement learning techniques towards bimanual tasks and introduces a language-conditioned behavioral cloning agent designed to learn coordinated 6-DoF dual-arm behaviors, addressing the lack of standardized simulated bimanual task suites [11]. Complementing this benchmark line, “Stabilize to Act” frames bimanual manipulation via role assignment (stabilizing vs. acting arm), explicitly reducing the effective complexity of coordination while improving robustness with limited demonstrations [12]. Recent hierarchical imitation-learning formulations also highlight the importance of decomposing bimanual tasks into intermediate structures, e.g., key pose-conditioned frameworks that separate high-level subgoals from low-level trajectory generation to balance coordination quality and inference speed [13]. Together, these works motivate cooperative manipulation formulations where feasibility is dominated by arm–arm–object coupling and workspace tightness, making constraint handling central when optimizing time-oriented objectives.

From a broader motion-planning viewpoint, modern pipelines increasingly combine optimization-based planning with learning components that warm-start or guide trajectory refinement in high-dimensional, constraint-heavy settings. A recent survey of optimization-based task and motion planning (TAMP) highlights how classical optimization methods are being integrated with learning-based components to improve scalability and robustness, especially under complex contact and environmental constraints [14]. At the motion-optimization level, recent work such as BOMP exemplifies this trend: trajectories are computed by optimization with explicit collision reasoning, while a neural network predicts an initialization to accelerate convergence and reduce computing time [15]. These advances are particularly relevant to dual-arm workspaces, where narrow feasible regions and coupled constraints can make purely local optimization sensitive to initialization and prone to stagnation, thereby motivating alternative global search strategies when objective functions are simulation-based or highly nonconvex.

When time optimality is a primary objective, recent literature increasingly moves beyond “pure retiming” toward approaches that jointly shape geometry and timing, often under obstacle, smoothness, and actuator limits. For example, Yu et al. propose time-optimal trajectory planning that searches the optimal path simultaneously rather than assuming a fixed geometric path, directly addressing limitations of methods that only optimize timing along a predefined route [16]. In a similar spirit, Wang et al. present a hybrid strategy (RRT + improved PSO) for time-optimal planning in a dynamic 3D environment while accounting for obstacle avoidance and smoothness, illustrating practical algorithmic hybrids to cope with complex constraints [17]. These lines of work reinforce that, in constrained scenes (typical in cooperative manipulation), time minimization becomes inseparable from feasibility preservation, and methods must explicitly prioritize collision-free coordination while improving execution time.

Because constrained trajectory optimization is often dominated by feasibility, modern optimization pipelines frequently adopt constraint-handling mechanisms that bias the search toward valid regions without delicate penalty tuning [18]. A recent comprehensive review by Rahimi et al. analyzes state-of-the-art constraint-handling techniques for population-based algorithms across single- and multi-objective settings, including feasibility rules, repair operators, and adaptive penalty strategies [16]. More recent work also discusses efficient treatments for implicitly constrained problems and highlights practical considerations that influence convergence and reliability in real applications [19]. These insights align naturally with feasibility-first (safety-first) designs in robotics, where collision-free behavior and coordination validity must be enforced before secondary objectives (e.g., time reduction) are optimized [5].

Bio-inspired metaheuristics remain attractive for robotics because they naturally accommodate nonconvex, discontinuous, and simulation-driven objectives, and can operate without analytic gradients. Recent surveys consolidate the state of the art and open issues for widely used optimizers such as PSO [20]. Newer metaheuristics also continue to emerge; for instance, the Gazelle Optimization Algorithm (GOA) has been widely adopted since its proposal and has shown competitive performance across benchmarks, motivating its use in robotics-oriented pipelines [21].

Overall, the literature suggests a clear opportunity at the intersection of (i) cooperative dual-arm bimanipulation under tight collision and coordination constraints, (ii) feasibility-first constraint handling to ensure safety and validity, and (iii) metaheuristic optimization to search effectively when the cost landscape is highly nonconvex and simulation-driven. This combination is particularly relevant when the objective is explicitly time-oriented (reducing execution time) while maintaining strict collision-free behavior in constraint-dominated dual-arm workspaces. This is the gap covered by this work.

## 3. Bio-Inspired Metaheuristic Algorithms

This paper employs three population-based, bioinspired optimizers—Particle Swarm Optimization (PSO), Whale Optimization Algorithm (WOA), and Gazelle Optimization Algorithm (GOA)—to solve the time-oriented, constraint-dominated trajectory planning problem introduced earlier. Although their biological metaphors differ, all three methods share the same computational backbone: a population of candidate solutions is iteratively refined through rules that alternate exploration (searching new regions) and exploitation (intensifying around promising solutions). In our framework, the algorithms interact with the planning problem exclusively through the objective function (including collision and feasibility penalties), which makes them well-suited to non-convex, non-smooth landscapes typical of collision-aware robotics [22].

### 3.1. Particle Swarm Optimization

Particle Swarm Optimization models collective decision-making in groups where individuals continuously adjust their motion by combining personal experience with social cues [23]. In PSO, each candidate solution is represented by a particle whose state is defined by its position $x_{i}^{t}$ and velocity $v_{i}^{t}$ at iteration t. The position update follows Equation (1), where the particle advances according to its current velocity.

(1) $$x_{i}^{t+1}=x_{i}^{t}+v_{i}^{t}$$

The key mechanism lies in how $v_{i}^{t+1}$ is updated. As expressed in Equation (2), the next velocity is formed by superimposing three effects: (i) an inertial component that preserves part of the previous motion, (ii) a cognitive attraction that pulls the particle toward its own best historical solution $p_{i}^{t}$, and (iii) a social attraction toward the best solution encountered by the swarm $g^{t}$. Random factors $r_{1}$ and $r_{2}$ introduce controlled variability, preventing the population from collapsing too early into suboptimal regions:

(2) $$v_{i}^{t+1}=\omega v_{i}^{t}+c_{1}r_{1}\left(p_{i}^{t}-x_{i}^{t}\right)+c_{2}r_{2}\left(g^{t}-x_{i}^{t}\right)$$

The memory terms $p_{i}^{t}$ and $g^{t}$ are updated using Equations (3) and (4). The personal best $p_{i}$ is replaced whenever a particle finds an improved solution, while the global best $g^{t}$ is computed as the best among all personal bests.

(3) $$p_{i}^{t+1}=\left\{\begin{array}{l} p_{i}^{t},\;\;\;\;\;\;\;\;\;\;\;\;\;\;\;\;if\;f\left(x_{i}^{t+1}\right)\geq f\left(p_{i}^{t}\right)\\ x_{i}^{t+1},\;\;\;\;\;\;\;\;\;if\;f\left(x_{i}^{t+1}\right)<f\left(p_{i}^{t}\right) \end{array}\right.$$

(4) $$g^{t}={argmin}_{i}f(p_{i}^{t})$$

**Figure 1:**
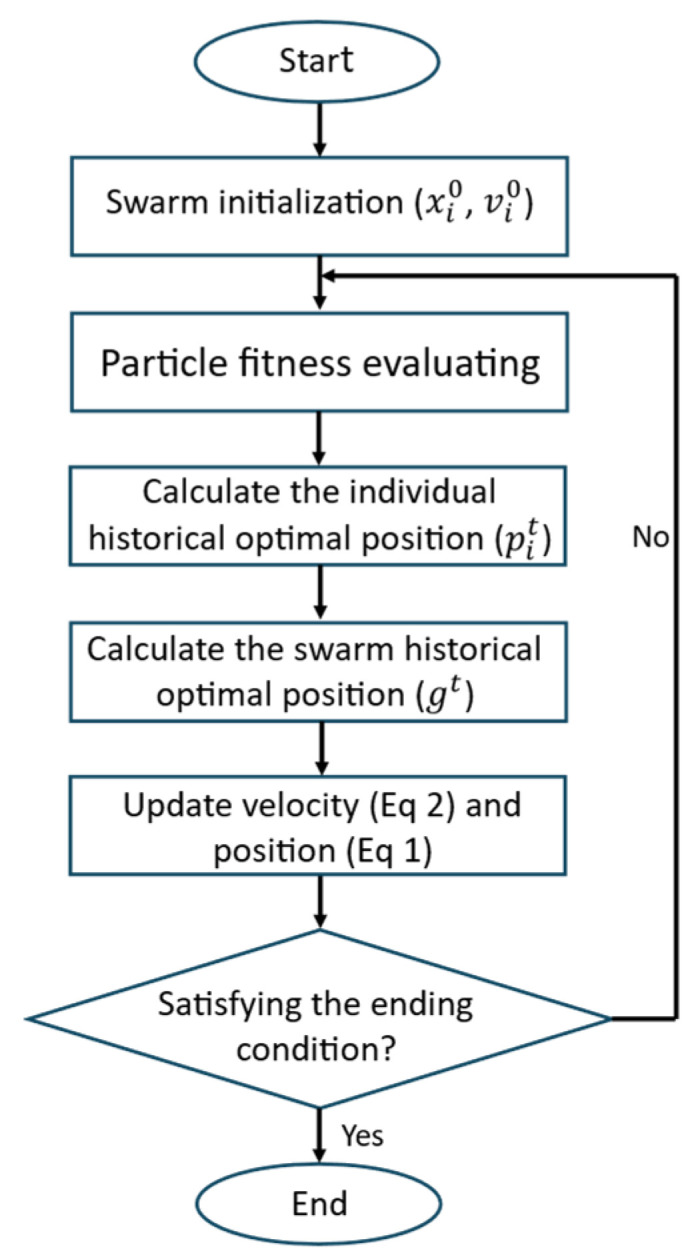
Particle Swarm Optimization Algorithm flowchart.

This procedure is summarized in Figure 1. This explicit memory structure makes PSO particularly effective as a baseline optimizer: it tends to converge rapidly when the objective presents a clear basin of improvement, while still retaining exploration through stochasticity in the velocity updates.

### 3.2. Whale Optimization Algorithm (WOA)

**Figure 2:**
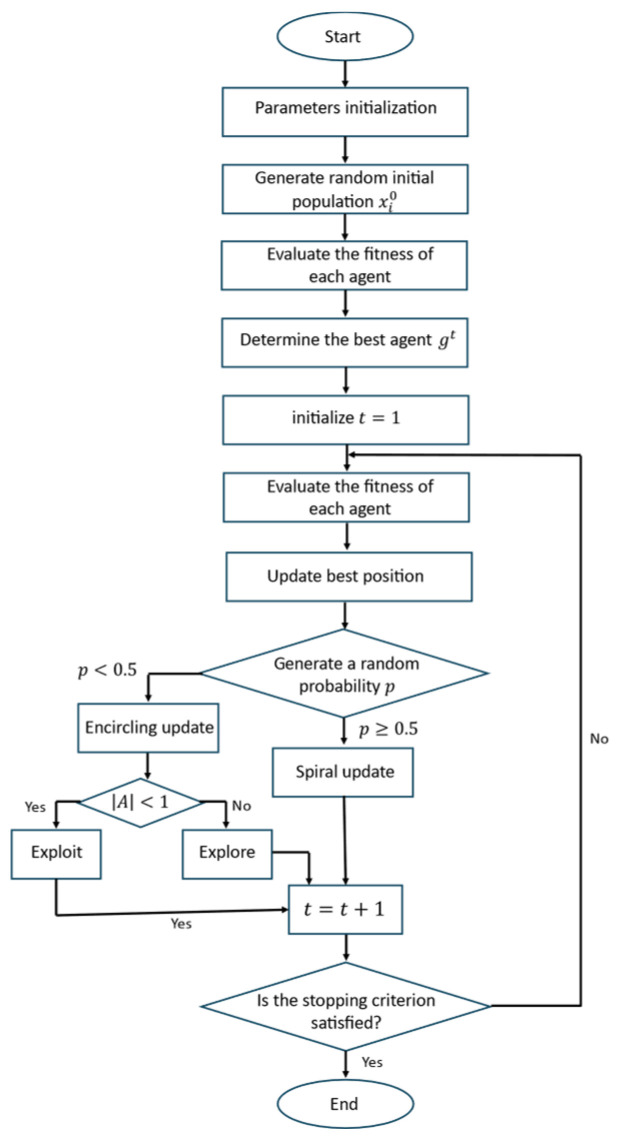
Whale Optimization Algorithm flowchart.

The Whale Optimization Algorithm emulates the hunting strategy of humpback whales, translating it into a two-mode search process that alternates between contraction toward a leader solution and spiral-shaped exploitation around it [24]. Each agent represents a candidate trajectory and updates its position relative to the best solution found so far, $g^{t}$. The algorithm flowchart is reported in Figure 2.

During the encircling (shrinking) phase, agents move toward the current best using Equation (5). The update is driven by coefficient vectors A and C, which modulate both the direction and the step size of the approach:

(5) $$x_{i}^{t+1}=g^{t}-A\times \left|C\times x_{i}^{{*}^{t}}-x_{i}^{t}\right|$$

A central feature of WOA is that the magnitude ∣A∣ acts as a switch between search regimes: values below unity emphasize exploitation near the leader, whereas values above or equal to unity promote exploration by encouraging agents to move away from the best-known area and probe alternative regions.

WOA complements this contraction mechanism with a spiral update that mimics the whale’s helical motion around prey. This behavior is expressed by Equation (6), which models a logarithmic spiral around the best solution:

(6) $$x_{i}^{t+1}=\left|g^{t}-x_{i}^{t}\right|\times e^{bl}\times \cos\left(2\pi l\right)+g^{t}$$

Here, b shapes the spiral and l∈[−1,1] introduces randomness. At each iteration, a probability parameter determines whether an agent applies the encircling update or the spiral update. This built-in alternation provides WOA with a practical balance: it can search broadly early on and then progressively concentrate around high-quality regions once feasibility is achieved.

### 3.3. Gazelle Optimization Algorithm (GOA)

The Gazelle Optimization Algorithm is motivated by the evasive locomotion of gazelles, characterized by sudden direction changes and agile transitions between wide-ranging movement and rapid refinement [21]. In GOA, each individual $x_{i}^{t}$ represents a candidate solution, and the update rule (Equation (7)) combines three terms: a stochastic displacement linked to the current “velocity” $v_{i}^{t}$, and a drift component guided by the local fitness landscape through $\nabla f(x_{i}^{t})$:

(7) $$x_{i}^{t+1}=x_{i}^{t}+r_{i}\cdot v_{i}^{t}+s_{i}\cdot \nabla f\left(x_{i}^{t}\right)$$

**Figure 3:**
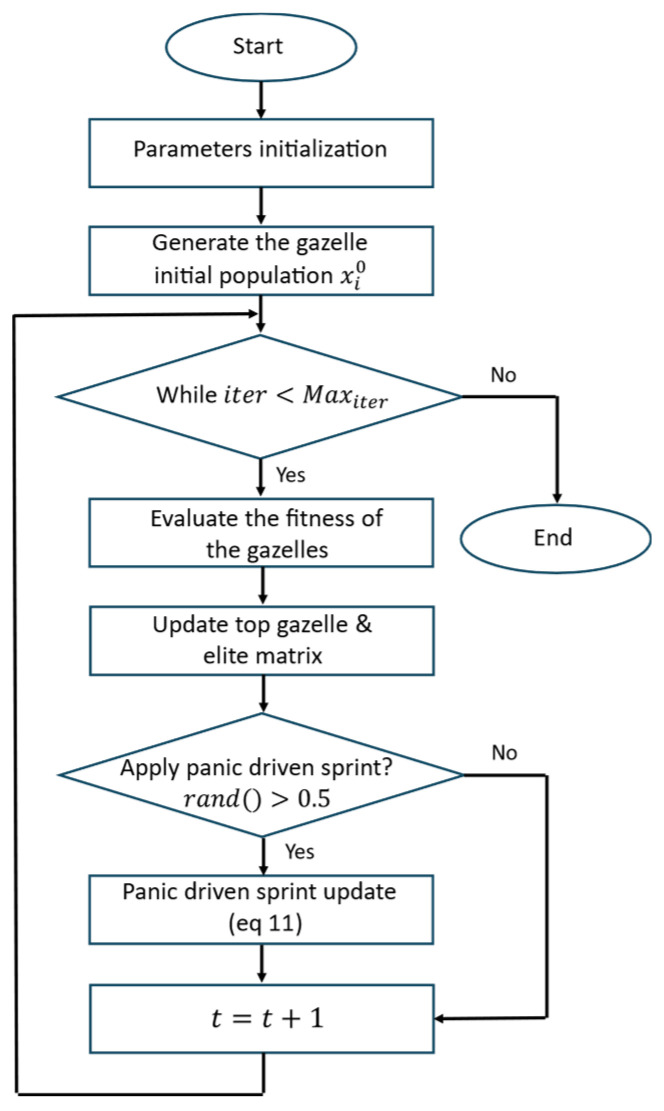
Gazelle Optimization Algorithm flowchart.

The coefficients $r_{i}$ and $s_{i}$ (Equations (8) and (9)) control the relative influence of randomness versus exploitation. Typically, $r_{i}$ injects variability through a scaled random factor, while $s_{i}$ decays over iterations so that the search gradually becomes more exploitative. The velocity term $v_{i}^{t}$ is calculated from recent displacement (Equation (10)), providing a form of short-term motion memory (Figure 3).

(8) $$r_{i}=\lambda \cdot rand(0,\;1)$$

(9) $$s_{i}=\eta \cdot e^{-\alpha t}$$

(10) $$v_{i}^{t}=x_{i}^{t}-x_{i}^{t-1}$$

To avoid stagnation, particularly common in rugged, constraint-heavy problems, GOA includes an additional perturbation (mutation) step (Equation (11)).

(11) $$x_{i}^{t+1}=x_{i}^{t+1}+\delta \cdot (rand(0,\;1)-0.5)$$

This operator deliberately “kicks” selected candidates away from their current region, encouraging renewed exploration when progress slows. The algorithm flowchart is depicted in Figure 3.

In practice, this makes GOA less prone to early clustering than strictly leader-driven methods, at the cost of additional hyperparameters and (when gradients are approximated numerically) potentially higher per-iteration computation. The baseline hyperparameters adopted for PSO/WOA/GOA are summarized in Appendix A to support reproducibility and to avoid case-specific tuning.

### 3.4. Qualitative Comparison and Suitability for Constrained Trajectory Planning

Although PSO, WOA, and GOA all belong to the same family of population-based metaheuristics, their search dynamics differ in ways that matter for collision-aware, time-oriented trajectory planning:

- PSO relies on explicit memory ($p_{i}^{t},\;g^{t}$) and smooth velocity updates, which often yield fast improvement once a feasible region is reached. However, if social influence dominates too strongly, the swarm can cluster prematurely around a suboptimal feasible basin.
- WOA alternates between contraction and spiral exploitation around the leader, while retaining a parameter-driven mechanism to force exploration when needed (∣A∣≥1). This structured switching can be advantageous in discontinuous landscapes where purely incremental updates struggle to escape poor regions.
- GOA blends stochastic motion, short-term velocity memory, and gradient-guided refinement, together with occasional perturbations to sustain diversity. This hybrid behavior is attractive when the optimizer must keep searching after feasibility is achieved, but it requires care when the objective includes non-smooth collision penalties, since gradient information can become unreliable unless properly smoothed or approximated.

From a computational standpoint, all three methods scale approximately with the product of population size and decision-space dimension, while the dominant cost in robotics typically comes from evaluating candidates (collision checks, kinematics, and trajectory timing). Consequently, the choice of optimizer is primarily justified by search behavior rather than arithmetic overhead: PSO offers a robust baseline, WOA provides a strong exploration–exploitation schedule through its dual update modes, and GOA contributes diversity-preserving dynamics that can reduce sensitivity to local minima.

In the next section, these algorithms are embedded into the same feasibility-first objective structure and evaluated under identical bounds and stopping criteria to enable a fair comparison in cooperative dual-arm bimanipulation.

## 4. Object-Centric Optimization of Dual-Arm Cooperative Trajectories

### 4.1. Dual-Arm System Model and Assumptions

**Figure 4:**
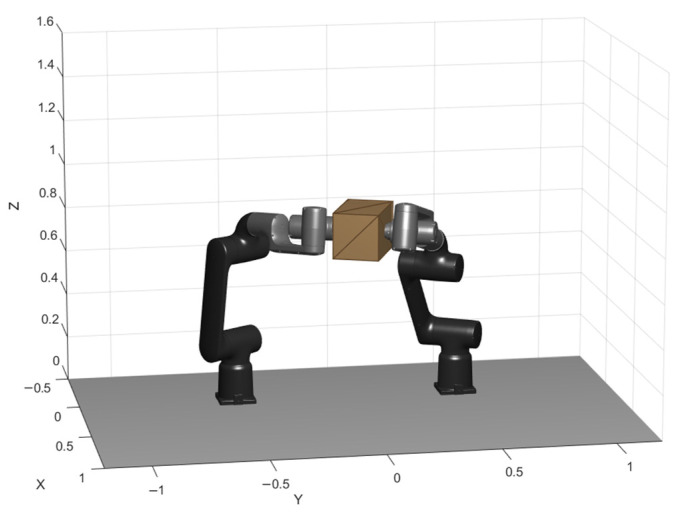
Dual-arm cooperative transport model.

This work considers a cooperative dual-arm manipulation setup in which two industrial manipulators jointly transport a single object while maintaining a stable bimanual grasp throughout the motion. The manipulated payload is a rigid box whose motion is planned in task space and executed by both arms in a coordinated manner. A schematic overview of the system is provided in Figure 4 to clarify the reference frame, the grasp locations on the object, and the geometric relationship between the two end-effectors during transport.

Each manipulator is modeled as a serial kinematic chain with $n_{L}$ and $n_{R}$ actuated joints, respectively, yielding a total of $n_{\mathrm{DoF}}=n_{L}+n_{R}$ controllable degrees of freedom for the dual-arm system. The object pose is described by a 6D configuration $p^{\mathrm{box}}=[x,\;y,\;z,\;\mathrm{roll},\;\mathrm{pitch},\;\mathrm{yaw}{]}^{⊤}\in SE(3)$, defined with respect to a fixed world frame. Cooperative transport is enforced by defining two grasp frames rigidly attached to the box at fixed offsets (left and right grasp points), such that the end-effector targets are uniquely determined by the box pose and these constant grasp transforms. This formulation effectively constrains the two arms to execute a synchronized motion consistent with a single object trajectory, preventing relative slip or regrasping during transport.

To focus the study on motion planning and kinematic feasibility, the following assumptions are adopted. (i) Rigid object and rigid grasp: the box is non-deformable and does not undergo internal shape changes; the grasp is maintained without slip, and the relative transform between each end-effector and the object remains constant over time. (ii) No loss of payload: the object does not fall, detach, or rotate freely, so the object’s pose is fully controlled through the cooperative kinematic references. (iii) Kinematics-only execution model: the analysis is restricted to kinematic constraints (reachability, joint limits, and collision avoidance). Dynamics, force/torque distribution between arms, frictional contact modeling, and compliance are outside the scope of this study. (iv) No physical pushing between robots: inter-arm interactions are treated purely as geometric constraints; direct arm–arm contact is considered infeasible and is prevented through explicit collision checking. (v) Static environment representation: obstacles are modeled as fixed bodies in the workspace during each motion plan evaluation.

Under these assumptions, the cooperative manipulation problem reduces to finding an object-centric trajectory in SE(3) that is simultaneously reachable by both arms, respects joint limits, and remains collision-free with respect to the environment, the payload, and inter-arm interference. The next sections build on this system description to define the decision variables, the cooperative kinematic pipeline that maps object motion to joint motion, and the collision-aware fitness function used within the optimization loop.

The objective of the optimization process is to generate a time-efficient and collision-free cooperative trajectory for a dual-arm robotic system transporting a shared object within a constrained workspace. The motion must satisfy task requirements defined by fixed initial and final configurations (e.g., pick and place poses) while respecting the physical and operational limits of both manipulators, including joint limits, reachability and safety constraints in the presence of surrounding obstacles.

### 4.2. Formulation of the Optimization Problem, Decision Variables and Constraints

**Figure 5:**
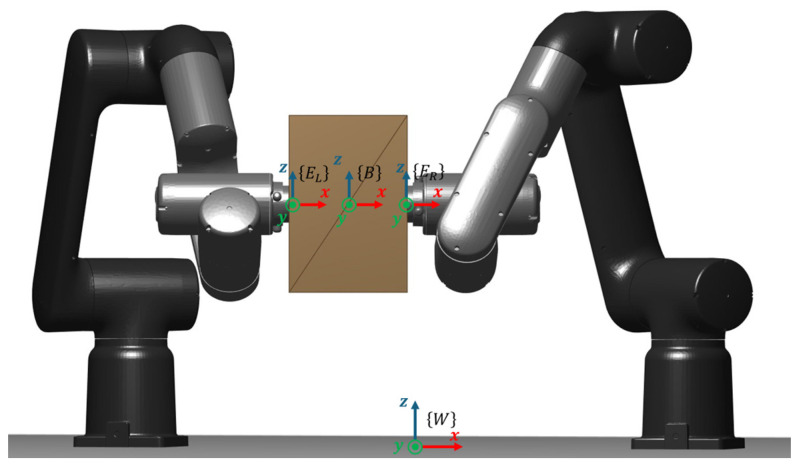
Object-centric waypoint mapping in the dual-arm cooperative transport model.

To pose the cooperative planning task as an optimization problem, the trajectory is defined in the task space of the transported object. The dual-arm system is commanded to manipulate a rigid box; therefore, the decision variables correspond to a set of intermediate box poses, $p^{\mathrm{box}},$ that parameterize the entire cooperative motion. Crucially, an object pose uniquely determines the Cartesian references of both end-effectors because the grasp geometry is assumed rigid and time-invariant. In other words, once a candidate box pose is specified, $p^{\mathrm{box}}$, the corresponding left and right grasp frames, $p^{L}\in SE(3)$, and $p^{R}\in SE(3)$, are obtained by applying fixed relative transforms (grasp offsets and orientations) from the box frame to each end-effector frame. This mapping converts an object-centric waypoint into two synchronized end-effector targets, one per robot, which are subsequently translated into joint configurations through the cooperative inverse-kinematics pipeline (Section 4.3). Figure 5 illustrates this geometric relationship: the box posed in the world frame, and the resulting end-effector reference poses for each manipulator.

Each adjustable waypoint, $p^{\mathrm{box}}=[x,\;y,\;z,\;\mathrm{roll},\;\mathrm{pitch},\;\mathrm{yaw}{]}^{⊤}\in SE(3)$, is defined by six parameters:

- $\left\{x,\;y,\;z\right\}$: Cartesian position of the box reference frame,
- $\left\{roll,,pitch,,yaw\right\}$: box orientation expressed through Euler angles.

The total number of optimization variables is given by Equation (12).

(12) $$n_{\mathrm{vars}}=6\;(n_{\mathrm{total}}-m)$$

Here, the trajectory is composed of $n_{\mathrm{total}}$ waypoints, among which m poses are fixed to satisfy task requirements (typically the initial and final poses, e.g., pick and place). The remaining $n=n_{\mathrm{total}}-m$ waypoints are optimized.

#### Constraints

A key aspect of the setup is how the position constraints were defined. In practice, reachability limits are naturally stated for each robot in the global workspace (e.g., admissible ranges in x,y,z for the end-effector or for a representative point on the tool). However, since the decision variables of the optimization are the object waypoints, these robot-centric constraints must be translated into constraints on the box pose. This translation is essential: a box waypoint is only admissible if it simultaneously induces end-effector poses that are reachable for both manipulators under the fixed grasp.

To formalize this mapping, where $\left\{W\right\}$ is the world frame, the object is expressed in Equation (13) by a homogeneous transform matrix.

(13) $$W_{T_{o}}=\left[\begin{array}{cc} W_{R_{o}} & W_{p_{o}}\\ 0 & 1 \end{array}\right]$$

where $W_{p_{o}}\in R^{3}$ is the object position and $W_{R_{o}}\in SO(3)$ its orientation. For each arm i∈{1,2}, the grasp is modeled as a constant homogeneous transform matrix, between the object frame and the end-effector frame (Equation (14)).

(14) $$T_{e_{i}}^{o}=\left[\begin{array}{cc} R_{e_{i}}^{o} & r_{i}^{o}\\ 0 & 1 \end{array}\right]$$

In this expression, $R_{e_{i}}^{o}\in SO(3)$ is the relative orientation and $r_{i}^{o}\in R^{3}$ is the relative position (offset) of the grasp point expressed in the object frame. Thus, the induced end-effector pose for arm i at any object pose is as shown in Equation (15).

(15) $$T_{e_{i}}^{W}=W_{T_{o}}\;T_{e_{i}}^{o}$$

If the workspace feasibility of arm i is expressed as an admissible set of end-effector positions (for instance, an axis-aligned box constraint), then

(16) $$W_{i}=\left\{p\in R^{3}\;|p_{i}\leq p\leq {\bar{p}}_{i}\right\}$$

Then, a candidate object waypoint is position-feasible only if the induced end-effector positions satisfy Equation (17).

(17) $$W_{e_{i}}=W_{p_{o}}+W_{R_{o}}r_{i}^{o},\;\;W_{e_{i}}\;\in \;W_{i},\;\;\;\;\;\;\;\;\;\;\;\;i=1,\;2$$

**Figure 6:**
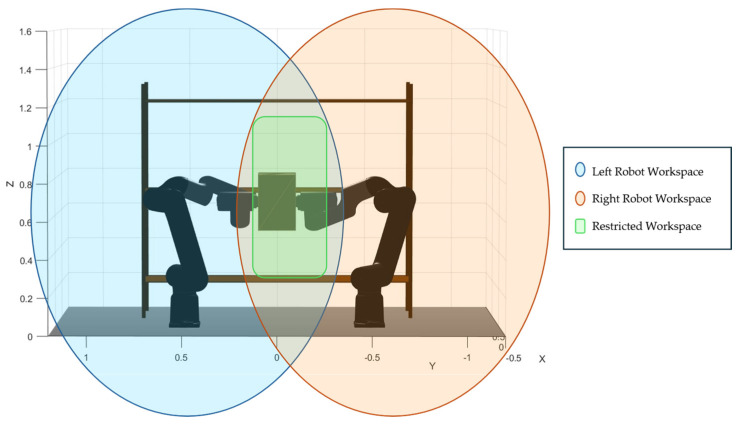
Reachability constraint mapping in the dual-arm cooperative transport model: individual end-effector workspace volumes and their intersection defining the admissible region for box waypoints.

Here, $W_{e_{i}}$ is the end-effector position of arm i induced by the object waypoint $\left(W_{p_{o}},W_{R_{o}}\right)$, and $r_{i}^{o}$ denotes the constant grasp offset expressed in the object frame. The constraint $W_{e_{i}}\in W_{i}$ enforces that each induced end-effector position lies within the admissible workspace set of the corresponding arm. Therefore, feasibility requires simultaneous satisfaction for i=1,2, which effectively maps robot-centric reachability limits into a single admissible region for the object pose. Figure 6 illustrates this idea visually. The shaded regions represent the individual reachable volumes associated with each arm, while the highlighted intersection region corresponds to the subset of space where the box pose can be placed without violating either arm’s reachability. In other words, the original (x, y, z) limits defined per robot are transformed into a single admissible region for the object, which is then used to bound the optimization variables and to reject infeasible waypoints during candidate evaluation.

Beyond reachability, the experimental setup also enforces standard constraints required in rack pick-and-place: (i) collision avoidance against the shelf, items, and ground; (ii) consistency with the rigid grasp; and (iii) boundary satisfaction at the initial and final object poses. Together, these constraints define a realistic, tightly constrained cooperative manipulation benchmark in which the optimizer must simultaneously ensure feasibility for both arms and improve trajectory efficiency.

### 4.3. Optimization Workflow and Fitness Function

**Figure 7:**
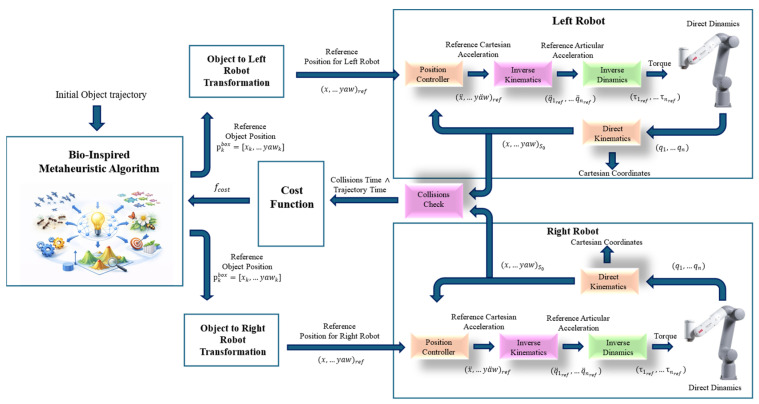
Bio-inspired metaheuristic optimization workflow for dual-arm cooperative manipulation.

The overall optimization workflow follows a closed-loop structure in which a bio-inspired metaheuristic algorithm iteratively proposes candidate solutions and receives performance feedback from the simulation-based evaluation. At each iteration, the optimizer generates a set of box waypoints (Equation (18)).

(18) $$p_{\mathrm{fixed},1}^{box},p_{1}^{box},\ldots ,p_{n}^{box},p_{\mathrm{fixed},m}^{box}$$

where the fixed poses enforce the task constraints and $p_{1}^{box},\ldots ,p_{n}^{box}$ are the decision variables. Each candidate waypoint set is then evaluated by the trajectory-generation and checking pipeline described in Section 4.3 and Section 4.4: the waypoints are converted into a smooth object motion, mapped to synchronized end-effector references through the rigid grasp model, transformed into joint-space configurations via cooperative inverse kinematics, and assessed for collisions along the sampled motion. This evaluation yields two scalar quantities for every candidate: the estimated execution time $t_{trajectory}$ and the accumulated collision time $t_{collision}$ (Section 4.4), which are combined into a single fitness value used to update the optimizer population and generate the next set of candidates. Figure 7 summarizes this iterative loop.

Candidate trajectories are evaluated with a fitness function that prioritizes safety over speed through a two-level decision rule. First, the trajectory is classified as feasible or infeasible based on the presence of collisions along the sampled motion. If any collision occurs, the solution is penalized in proportion to the accumulated collision time. Only when the candidate is entirely collision-free is execution time used as the optimization objective.

This staged behavior is implemented as shown in Equation (19):

(19) $$f_{c}=\left\{\begin{array}{cc} 1+t_{\mathrm{collision}}, & \mathrm{if}\;t_{\mathrm{collision}}>0\\ \frac{t_{\mathrm{trajectory}}}{k}, & \mathrm{if}\;t_{\mathrm{collision}}=0 \end{array}\right.$$

where $t_{\mathrm{collision}}$ is the total time in collision, $t_{\mathrm{trajectory}}$ is the estimated execution time, and k is a normalization constant selected to scale time-based costs to a comparable range (set to k=50 s in this work). With this definition, any colliding trajectory yields $f_{c}>1$, while collision-free candidates produce $f_{c}<1$ if their duration remains below k. Consequently, optimization naturally focuses on discovering feasibility first and then refining the motion to reduce execution time within the feasible set.

To select a suitable value of k for a new application, a practical approach is to estimate a conservative upper bound on feasible execution time by simulating motions across the extremes of the workspace with reduced speed and then rounding up to a safe margin. This choice keeps the fitness scale stable across scenarios and ensures a clear separation between infeasible and feasible solutions.

### 4.4. Control and Trajectory Generation for Cooperative Dual-Arm Manipulation

Given the optimizer output $p_{k}^{box}=[x_{k},y_{k},z_{k},roll_{k},pitch_{k},yaw_{k}]$, and the constrained fixed waypoints, {$p_{\mathrm{fixed},1}^{box},p_{\mathrm{fixed},2}^{box},\ldots ,p_{\mathrm{fixed},m}^{box}\}$, the box path is converted into a continuous trajectory $T_{box}\left(t\right)\;$ using a smooth polynomial interpolator (quintic in this work). This interpolation enforces continuity in position, velocity, and acceleration, avoiding sharp discontinuities that would be incompatible with industrial execution. The resulting discretized trajectory is sampled uniformly, producing a sequence of box poses ${\left\{T_{box}\left(t_{i}\right)\right\}}_{i=1}^{N}$ used for inverse kinematics and collision evaluation.

#### 4.4.1. From Box Pose to Left/Right End-Effector References

For each sampled time instant $t_{i}$, the box pose is expressed as a homogeneous transform (Equation (20)).

(20) $$T_{box}(t_{i})=\left[\begin{array}{cc} R_{box}(t_{i}) & p_{box}(t_{i})\\ 0^{⊤} & 1 \end{array}\right]$$

where $p_{box}\in R^{3}$ and $R_{box}\in SO(3)$ is obtained from the box roll–pitch–yaw angles.

The cooperative grasp is imposed by defining two fixed grasp offsets in the box frame, separated laterally by a distance d. These offsets are mapped into the world frame through the box rotation represented in Equations (21) and (22).

(21) $$p_{L}(t_{i})=p_{box}(t_{i})+R_{box}(t_{i})\;[+d,\;0,\;0{]}^{⊤}$$

(22) $$p_{R}(t_{i})=p_{box}(t_{i})+R_{box}(t_{i})\;[-\;d,0\;,\;0{]}^{⊤}$$

**Figure 8:**
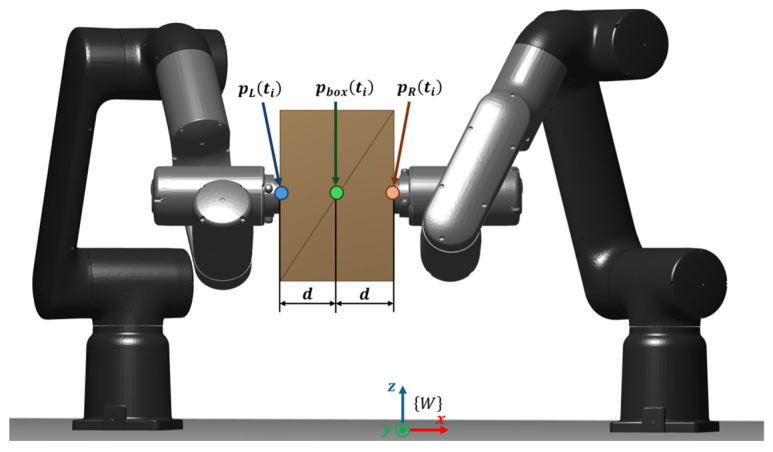
Schematic mapping from the box pose to the left/right end-effector position references $p_{L}(t_{i})$ and $p_{R}(t_{i})$ using fixed grasp offsets.

To further comprehend these Equations (23) and (24), a schematic representation is shown in Figure 8.

In addition, the end-effector orientations are defined as rigidly attached to the object through constant relative rotations $R_{L,rel}$ and $R_{R,rel}$:

(23) $$R_{L}(t_{i})=R_{box}(t_{i})\;R_{L,rel},R_{R}(t_{i})=R_{box}(t_{i})\;R_{R,rel}$$

This produces two time-varying target homogeneous transforms matrices $T_{L}(t_{i})$ and $T_{R}(t_{i})$, which become the Cartesian references for each manipulator. As a result, the dual-arm system follows a synchronized motion that preserves the object grasp geometry across the whole trajectory.

#### 4.4.2. Time Parametrization

Trajectory timestamps are assigned based on the cumulative Euclidean distance travelled by the box position. Assuming a constant nominal box speed v, the time sequence is computed as:

(24) $$t_{0}=0,t_{i}=t_{i-1}+\frac{∥p_{box}(t_{i})-p_{box}(t_{i-1})∥}{v}$$

This provides a consistent execution-time estimate for each candidate trajectory and directly links the optimized object path length to the resulting motion duration.

### 4.5. Collision Evaluation

To guarantee trajectory feasibility, collision assessment is performed at evaluation time for every candidate solution generated by the optimizer. Once the continuous motion $T_{box}(t)$ is obtained, it is sampled over time to produce a finite set of configurations ${\left\{q_{L}(t_{i})\right\}}_{i=1}^{N}$ and ${\{q_{R}(t_{i})\}}_{i=1}^{N}$ (from the cooperative IK pipeline) and a corresponding set of box poses ${\left\{T_{box}(t_{i})\right\}}_{i=1}^{N}$. Collision status is then evaluated at each sample, and the collision time is approximated by accumulating the time intervals associated with samples flagged as colliding.

The collision engine follows a pairwise intersection paradigm between convex shapes. Obstacles in the workspace are represented as a set $\omega_{O}=\{O_{1},\ldots ,O_{N_{O}}\}\subset R^{3}$, where each $O_{i}$ is a convex body (either an analytical primitive or a convex component resulting from mesh decomposition). At each sample, the algorithm checks intersections between: (i) robot links and $\omega_{O}$, (ii) left-arm links and right-arm links (inter-arm interference), and (iii) the box volume and $\omega_{O}$. This yields three complementary feasibility conditions that jointly prevent unsafe solutions (robot hitting obstacles), cooperative incompatibilities (arm–arm contact), and payload collisions (object intersecting the environment).

Intersection tests rely on the Gilbert–Johnson–Keerthi (GJK) method, which determines whether two convex sets A and B overlap by reasoning in the space of their Minkowski difference:

(25) A⊖B={ a−b ∣ a∈A, b∈B }

A collision exists precisely when the origin belongs to that difference set:

(26) A∩B≠Ø⇔0∈A⊖B

Here, 0 denotes the zero vector in Minkowski space (not the workspace origin). In practice, GJK iteratively builds a simplex (point/segment/triangle/tetrahedron) using support points of A⊖B and progressively refines it to decide whether the simplex can enclose 0. If the origin can be enclosed, the two convex bodies intersect; otherwise, the algorithm converges to a separating condition and reports no collision.

In this work, collision checking is implemented in three stages per sample:

1. Robot–obstacle collisions: each robot link is tested against every element in $\omega_{O}$. Self-collisions are ignored in this stage to focus on interactions with the environment.
2. Inter-arm collisions: self-collision checking is enabled and only contacts involving one body from the left chain and one from the right chain are treated as critical, since these correspond to true cooperative interference.
3. Box–obstacle collisions: the transported object is modeled as a collision volume whose pose is updated using $T_{box}(t_{i})$ and tested against $\omega_{O}$, ensuring that the payload remains collision-free even when the manipulators themselves are feasible.

A binary collision indicator $c_{i}\in \{0,1\}$ is stored for each time sample, aggregating the three collision conditions above. The total collision time is then estimated by a discrete integral:

(27) $$t_{collision}\approx \sum_{i=2}^{N}c_{i}\;(t_{i}-t_{i-1})$$

This procedure provides a consistent feasibility metric within the optimization loop, while remaining computationally tractable for repeated evaluation across thousands of candidate trajectories.

**Figure 9:**
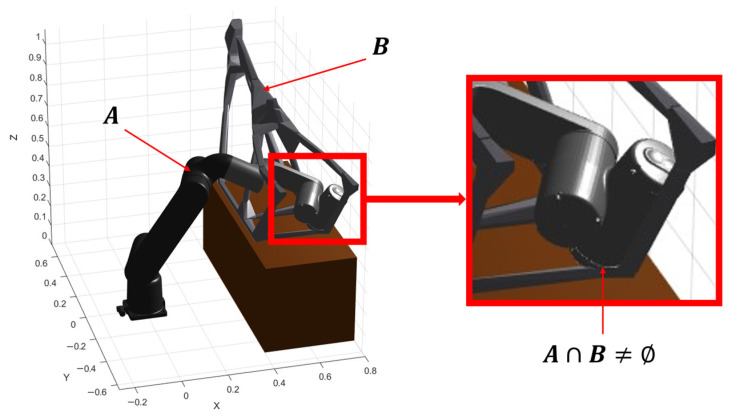
GJK-based collision detection example during trajectory evaluation: intersection between a robot link (set A) and an obstacle (set B); the zoomed view highlights the contact region.

Figure 9 shows a real collision detected during the optimization process. The robot link involved in contact corresponds to set A, while the motorcycle chassis corresponds to set B. The magnified region highlights the exact location where the intersection occurs, i.e., where the Minkowski difference A⊖B contains the origin.

## 5. Use Case: Rack Pick-And-Place by Dual Robot CRB15000 Optimization

The proposed framework was validated on a representative rack pick-and-place task in which a dual-arm robotic system cooperatively transports a shared box within a cluttered shelf environment. The task emulates a common industrial manipulation situation: the payload must be moved from a well-defined pick pose to a target place pose while maintaining a stable bimanual grasp and avoiding collisions with the shelf structure, stored items, and the ground plane. This setting is particularly informative because feasibility is not only dictated by obstacle avoidance, but also by the closed-chain coordination imposed by the two manipulators grasping the same rigid object.

### 5.1. Dual-Arm Robotic Setup

**Figure 10:**
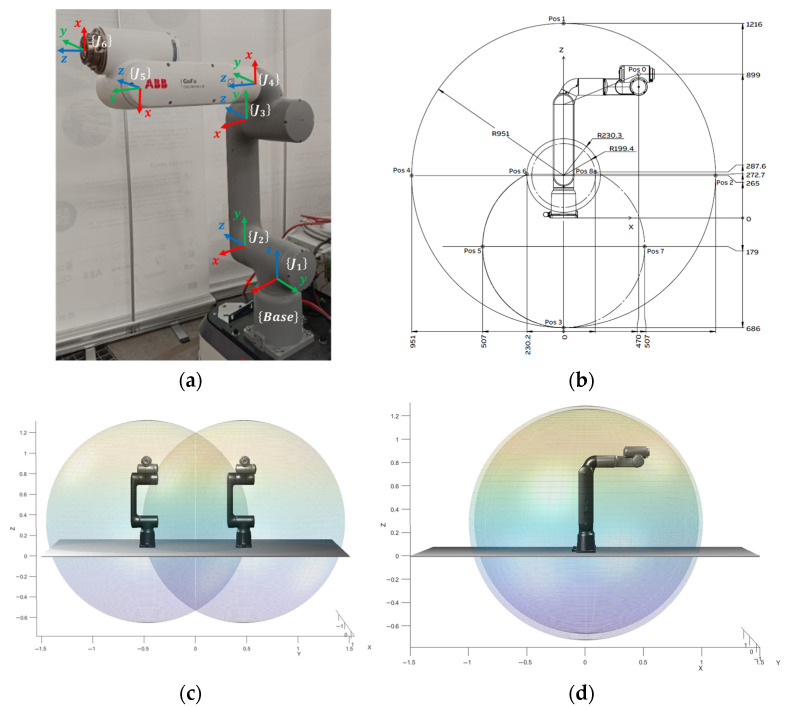
GoFa 5 Workspace: (**a**) ABB GoFa 5 manipulator with frames. (**b**) Range measurement visualization [25]. (**c**) Bimanual robot range (front-view). (**d**) Bimanual robot range (side-view).

The validation was conducted with a dual-arm cooperative setup based on two ABB GoFa 5 collaborative manipulators (CRB 15000/95), placed on opposite sides of a shelf rack to transport a shared rigid box under a fixed bimanual grasp. Each GoFa 5 is a 6 DOF (6-axis) robot with 5 kg payload, 950 mm reach (wrist)/1050 mm reach (flange), and 0.02 mm pose repeatability, enabling precise cooperative motions in constrained environments. The platform also supports fast end-effector motions, with a maximum TCP speed of 2.2 m/s and a maximum TCP acceleration (controlled motion, nominal load) of 36.9 m/s^2^, which is relevant for time-efficient trajectory execution once feasibility is achieved. The robot kinematic structure and the reachable workspace used in this study are summarized in Figure 10.

**Figure 11:**
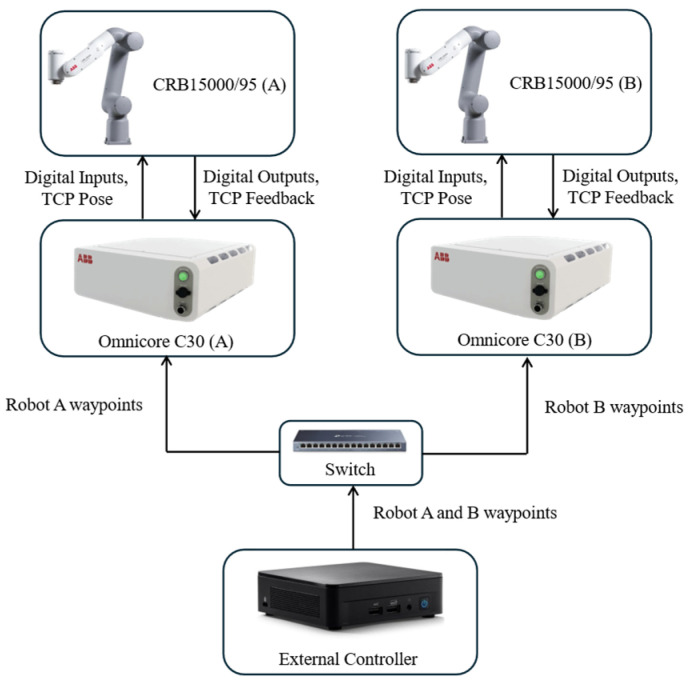
Dual-arm control architecture.

Figure 11 summarizes the controller architecture of the dual-arm platform. Each GoFa 5 manipulator is driven by an individual OmniCore C30 controller, while a supervisory external PC generates the planned setpoints for both arms and sends them through an Ethernet network (via a switch) to each controller. The OmniCore units execute the low-level joint motion control and return robot-state feedback to the external PC for logging and evaluation.

### 5.2. Work-Cell Layout and Rack Environment Geometry

The two robots are placed facing the rack from opposite sides. The distance from each robot base to the rack front plane is 0.5 m, and the distance between robot bases is 1.0 m (measured between base-frame origins). This arrangement produces a substantial shared workspace in front of the rack, while still imposing strong geometric constraints due to the limited clearances between shelves.

The rack is modelled as a static structure represented by collision bodies (shelves and frame elements), and a ground plane is included as a collision object. The following dimensions are fixed across all experiments:

- Shelf heights (from the ground plane): 0.20 m, 0.60 m, and 1.00 m.
- Rack footprint: 1.00 m (length) × 0.40 m (depth).

These dimensions define the key spatial limitations of the benchmark, since shelf clearances and rack depth restrict feasible object orientations and intermediate waypoints during cooperative transport.

**Figure 12:**
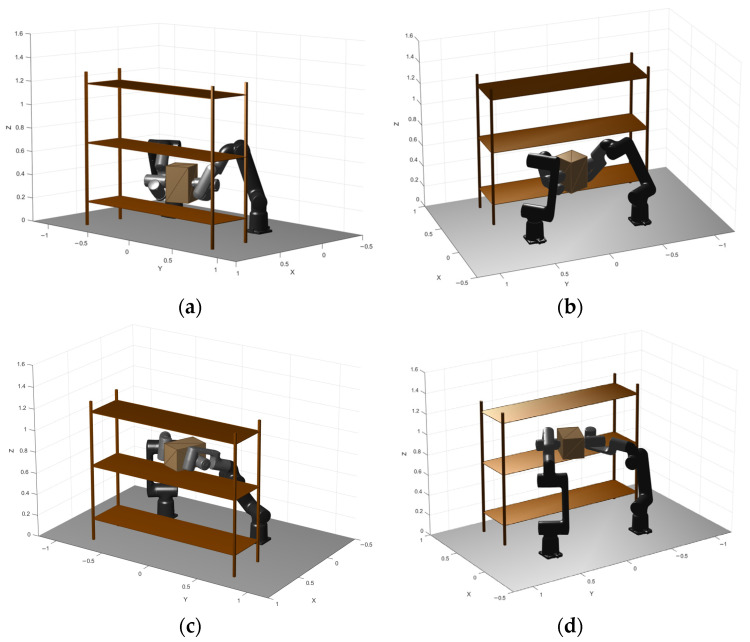
Experimental setup: (**a**) Frontal isometric view of pick position; (**b**) back isometric view of pick position; (**c**) frontal isometric view of place position; (**d**) back isometric view of place position.

Figure 12 depicts the experimental work cell and the cooperative manipulation configuration. The experiment is defined by two task states: pick (Figure 12a,b) and place (Figure 12c,d), both expressed in the global workspace frame. These two poses serve as boundary conditions for the trajectory generator, and all candidate solutions produced by the optimizer are required to start and end exactly at these poses.

### 5.3. Metrics

Comparing PSO, WOA, and GOA under the same experimental conditions, a set of evaluation metrics is defined to capture three complementary aspects of performance: feasibility discovery, convergence behavior, and solution quality. Using the same metrics across all use cases enables a fair, reproducible assessment and allows the results to be reported in a unified comparative table. The metrics used in this study are:

(a) Iterations to First Feasible Solution

IFFS counts how many optimization iterations are needed until an algorithm produces its first collision-free trajectory. Because the search typically starts from randomly generated candidates, many of which are infeasible, this indicator reflects how quickly the method can escape infeasible regions and reach the feasible set. Smaller IFFS values imply earlier feasibility discovery, which is especially relevant in industrial settings where an initial valid plan may be required quickly.

(b) Time to First Feasible Solution (TFFS).

TFFS measures the elapsed wall-clock time (in seconds) required to obtain the first collision-free trajectory. While IFFS focuses purely on iteration count, TFFS additionally captures the computational cost per iteration. This is important because PSO often executes lightweight updates and can perform many iterations within the same runtime, whereas WOA and GOA may incur higher per-iteration overhead. Lower TFFS values indicate faster feasibility in real time.

(c) Best Cost Value (BCV).

BCV is the lowest value reached by the fitness function during the optimization run. It summarizes the best solution found according to the staged objective (collision avoidance first, then time minimization).

(d) Trajectory Execution Time (TET).

TET is the simulated duration (in seconds) of the motion corresponding to a collision-free solution. This metric directly quantifies operational efficiency: among feasible trajectories, smaller TET values correspond to faster executions (and typically smoother, more kinematically efficient motions under the same interpolation and control pipeline).

(e) Iteration Throughput (IT).

IT represents the number of iterations completed per hour of runtime. This metric is reported to contextualize convergence results under a fixed time budget, since algorithms differ in how expensive each iteration is. Higher IT indicates that the method can explore more candidate solutions within the allowed computation time.

### 5.4. Simulation Environment and Evaluation Workflow

PSO, WOA, and GOA were run in a Matlab 2024b environment under identical bounds and stopping conditions, and the resulting trajectories were assessed in terms of feasibility discovery, convergence behavior, and execution-time efficiency. Note that this choice of environment is not restrictive: the proposed framework is simulation-platform-agnostic and can be integrated into any robotics simulation stack that provides standard motion-evaluation primitives, such as forward kinematics and collision checking. It should be noted that the simulation environment is purely kinematic; no contact dynamics, friction models, or restitution effects are considered, as grasping is assumed to be ideal and rigid.

**Figure 13:**
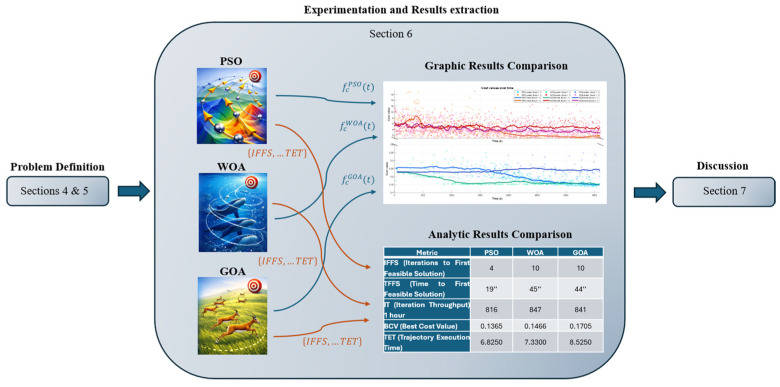
Schematic overview of the validation process and results reporting in Section 6.

**Figure 14:**
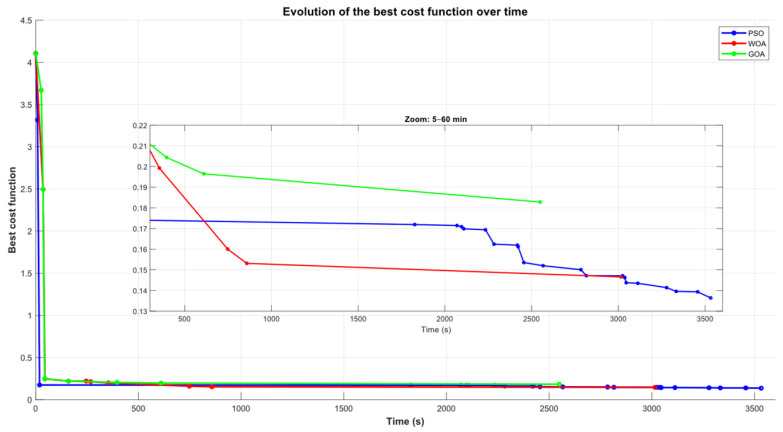
Convergence of the best cost function value across optimization iterations for PSO (blue), WOA (red), and GOA (green); inset highlights iterations 400–1000.

**Figure 15:**
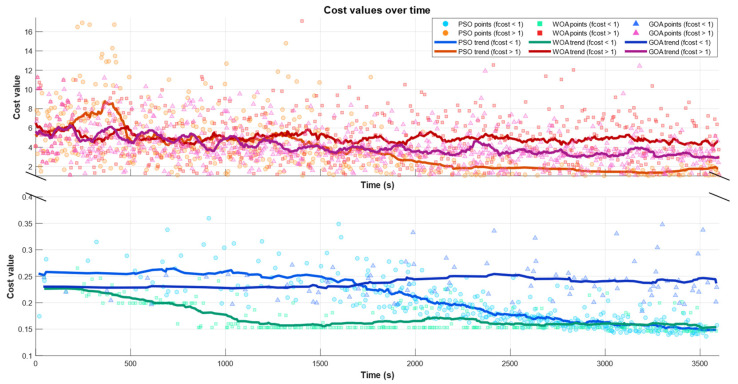
Cost-function scatter of all evaluated candidates over iterations (PSO/WOA/GOA), split into infeasible ($f_{\cos t}>1$) and feasible ($f_{\cos t}<1$) regimes.

**Figure 16:**
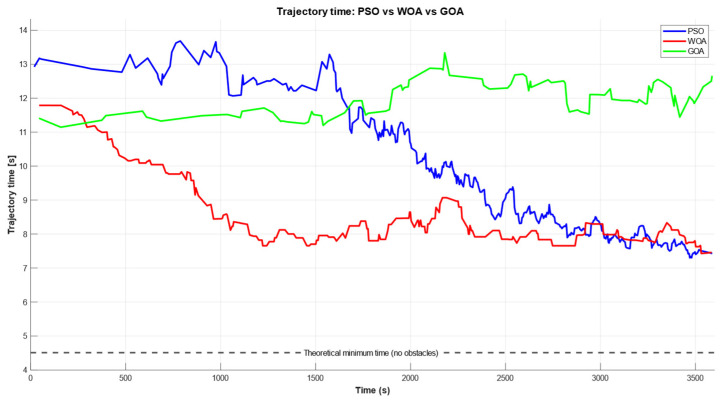
Trajectory execution time estimated from collision-free candidates for the three optimization algorithms (PSO, blue; WOA, red; GOA, green).

The proposed approach and the evaluation workflow are summarized in Figure 13. Following this scheme, each optimizer produces candidate trajectories that are assessed through the staged cost function and the selected performance metrics. The results are then reported as: (i) the cost-function evolution and feasibility trends (Figure 14 and Figure 15), (ii) the execution-time evolution of the collision-free subset (Figure 16), (iii) the qualitative comparison of the optimized trajectories and their motion sequences in the rack workspace (Figure 19, Figure 20 and Figure 21), (iv) the robot forces during the trajectory (Figure 22), and (v) the consolidated metrics comparison (Table 1).

## 6. Discussion of Results

### 6.1. Evolution of the Fitness Function

Figure 14 reports the evolution of the best cost value over the optimization iterations. A common pattern emerges across the three optimizers: the cost collapses sharply at the beginning, indicating that feasible solutions lie relatively close to the initial sampling distribution and can be reached early in the search. The key differences appear after this first feasible region is found. As optimization progresses, the curves separate and reveal distinct refinement dynamics. The zoomed window (iterations 400–1000) makes this particularly clear: PSO (blue) continues to introduce stepwise improvements, steadily pushing the solution toward lower-cost basins, whereas WOA (red) improves more gradually and GOA (green) remains consistently higher, with comparatively limited gains at later iterations. This late-stage behavior anticipates the final ranking of best solutions: PSO ultimately reaches the lowest objective value (BCV = 0.1365), followed by WOA (0.1466) and GOA (0.1705), as summarized in Table 1.

Figure 15 further details the optimization dynamics by plotting the entire population of evaluated solutions over time, explicitly separating collision-free candidates $f_{\cos t}<1$ from colliding ones $f_{\cos t}>1$ or each optimizer (PSO in blue, WOA in green, and GOA in magenta). The upper panel shows the distribution of colliding samples $f_{\cos t}>1$, where cost values remain in a high range and exhibit limited improvement. The lower panel reports the feasible region $f_{\cos t}<1$, where solutions concentrate within a narrow low-cost band and the refinement process takes place. In both panels, markers represent individual evaluated particles/agents, while the thick solid curves depict the smoothed trend of the population. The broken *y*-axis is used to emphasize the large numerical gap between the colliding and collision-free regimes and to improve the readability of late-stage improvements in the feasible band.

The trend of the feasible subset clearly differentiates the optimizers: PSO exhibits the steepest and most sustained decrease in the $f_{\cos t}<1$ trend, indicating consistent late-stage improvement once feasibility is reached; WOA also reduces the feasible cost over time but with a milder slope, suggesting slower exploitation; and GOA shows the weakest improvement, with a comparatively flat feasible trend that aligns with its higher best-cost values. Overall, this distribution-level view reinforces the convergence curves by showing that PSO not only attains the best final solution but also maintains a stronger refinement tendency within the collision-free basin, while WOA and especially GOA display progressively less effective improvement in the feasible region.

Figure 16 translates the collision-free optimization outcomes into an execution-oriented metric by mapping the feasible subset $f_{cost}<1$ into the corresponding trajectory execution time. In this view, lower values indicate faster motions, and the curves reveal how each optimizer improves time efficiency as the search progresses over the one-hour budget. WOA (red) exhibits the earliest rapid decrease, reaching a low-time basin relatively quickly, while PSO (blue) shows a more delayed but stronger late-stage refinement, eventually converging to the best final execution time. In contrast, GOA (green) remains comparatively flat and higher throughout the run, indicating limited improvement in time efficiency once feasibility is achieved. The dashed line represents the minimum theoretical time obtained from the straight-line translation between the pick and place positions (ignoring obstacles and reorientation), highlighting the performance gap imposed by the rack constraints and the required cooperative manipulation.

### 6.2. Performance Comparison

Table 1 consolidates the performance in terms of feasibility, search efficiency, and motion efficiency under the hyperparameter settings reported in Appendix A. First, PSO demonstrates a clear advantage in reaching feasibility quickly, requiring only 4 iterations to obtain the first feasible solution (IFFS) and 19 s in wall-clock time (TFFS). By comparison, WOA and GOA require 10 iterations and approximately 44–45 s, respectively, before producing a feasible trajectory. Second, the iteration throughput over one hour is comparable across methods (816–847 iterations), suggesting that the observed performance differences stem primarily from the search dynamics and solution quality, not from computational speed. Finally, the most relevant outcome for the rack pick-and-place task—how fast the robot can execute the resulting motion—follows the same ordering: PSO yields the shortest execution time (TET = 6.825 s), WOA is slightly slower (7.330 s), and GOA produces the slowest motion (8.525 s). Metaheuristic performance can be sensitive to hyperparameter choices; therefore, while Appendix A provides the configuration used to ensure a fair and reproducible comparison, different parameterizations may lead to quantitatively different outcomes.

**Table 1:** Performance metrics.

Metric	PSO	WOA	GOA
IFFS (Iterations to First Feasible Solution)	4	10	10
TFFS (Time to First Feasible Solution)	19″	45″	44″
IT (Iteration Throughput) 1 h	816	847	841
BCV (Best Cost Value)	0.1365	0.1466	0.1705
TET (Trajectory Execution Time)	6.8250	7.3300	8.5250

Overall, the results indicate that, in this rack-constrained pick-and-place scenario, PSO not only reaches feasibility earlier but also sustains improvement during the refinement phase, translating into trajectories with lower cost and shorter execution time. WOA remains competitive and reliably convergent, albeit to slightly less time-efficient motions, while GOA exhibits more limited late-stage refinement under the same optimization budget.

### 6.3. Analysis of the Optimized Trajectories

**Figure 17:**
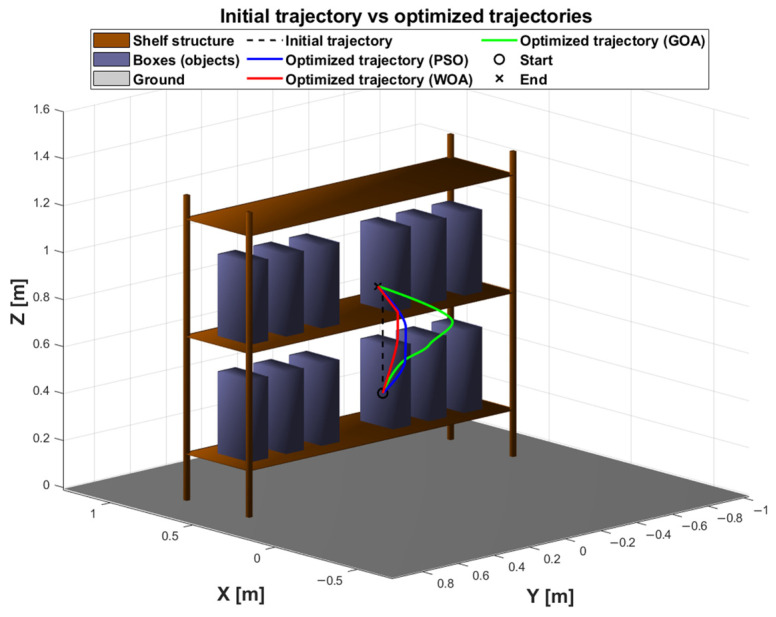
Comparison between the initial trajectory and the trajectories optimized by PSO (blue), WOA (red), GOA (green).

Figure 17 complements this convergence view by showing what these numerical differences mean in the workspace itself. The initial trajectory provides a feasible reference but does not fully exploit the available clearance within the rack. In contrast, each optimizer reshapes the path to remain collision-free while shortening and smoothing the motion through free space. Among them, the PSO trajectory appears the most direct and compact, with fewer detours around the stored objects; WOA produces a similarly feasible motion but with a slightly longer route; and GOA yields the least time-efficient motion, reflecting a more conservative or less refined passage through the constrained region. This qualitative ordering is consistent with the cost curves in Figure 13 and becomes even more tangible when considering execution time.

**Figure 18:**
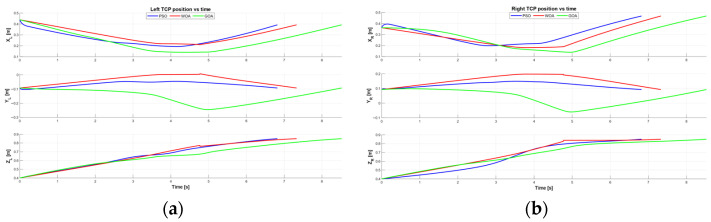
TCP Cartesian coordinates versus time for the optimized dual-arm execution. (**a**) Left-arm TCP position $\left(x_{L},y_{L},z_{L}\right)$ versus time for PSO (blue), WOA (red), and GOA (green). (**b**) Right-arm TCP position $\left(x_{R},y_{R},z_{R}\right)$ versus time for PSO (blue), WOA (red), and GOA (green).

To further support the verification of the obtained solutions, Figure 18 reports the time evolution of the Cartesian TCP position for both manipulators during the optimized execution. Specifically, Figure 18a shows the left-arm TCP coordinates $\left(x_{L},y_{L},z_{L}\right)$ versus time, and Figure 18b shows the right-arm TCP coordinates $\left(x_{R},y_{R},z_{R}\right)$ versus time, for the best trajectories found by PSO, WOA, and GOA. These plots provide an execution-oriented view of the motion, making it easier to compare how each optimizer shapes the cooperative trajectory over time and to verify that the resulting motions are smooth and consistent with the constrained rack manipulation.

**Figure 19:**
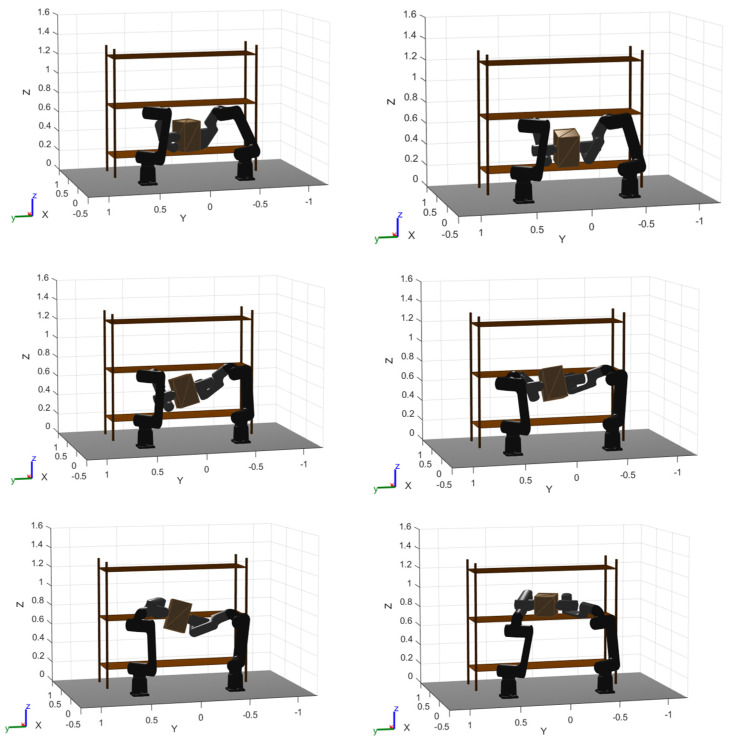
PSO optimized trajectory movement. Pivoted reorientation with right-arm anchoring (left-to-right support transfer).

**Figure 20:**
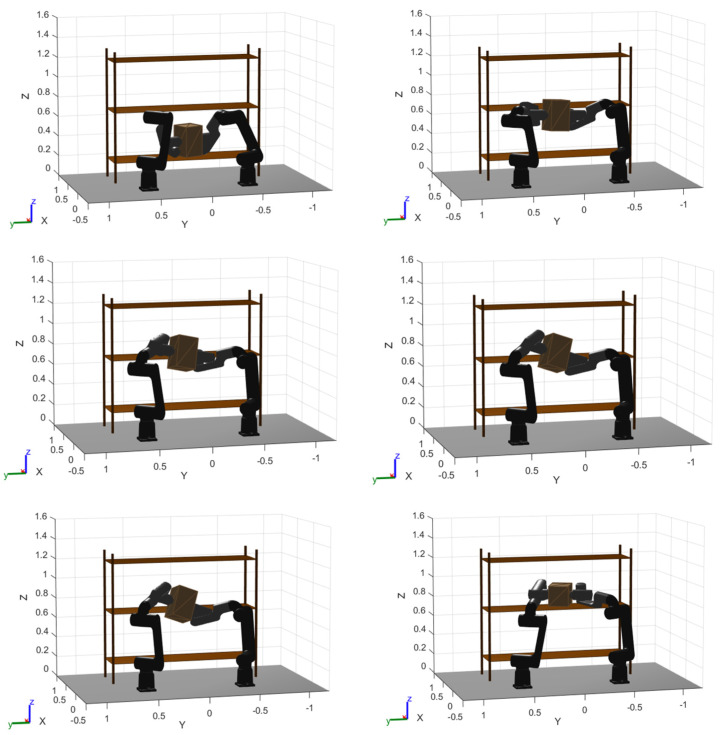
WOA optimized trajectory movement. Clearance-first vertical lift followed by right-arm pivot rotation.

**Figure 21:**
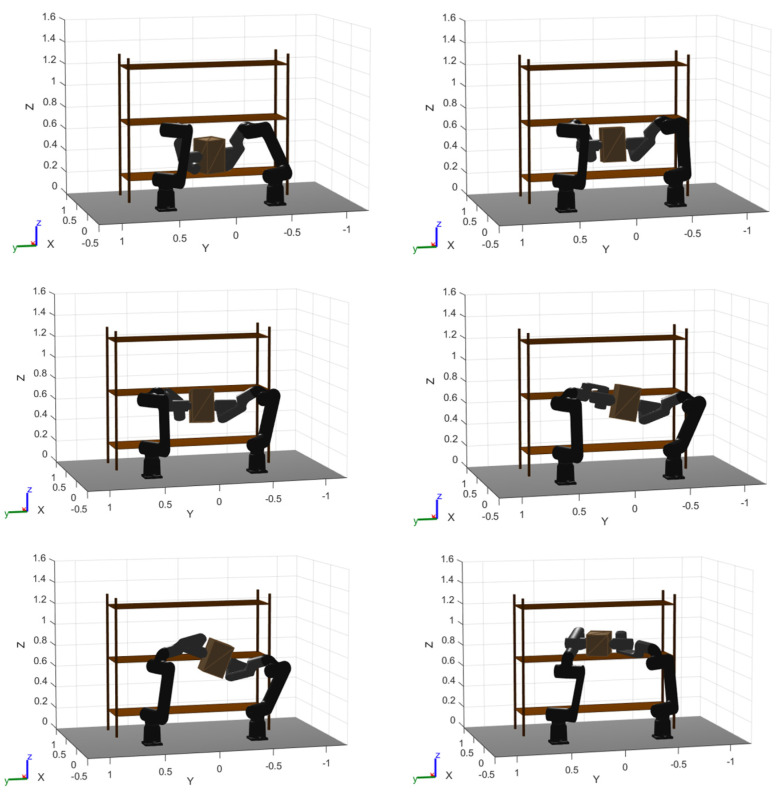
GOA optimized trajectory movement. Conservative wide-swing transfer with distributed reorientation in a constrained aisle.

To complement Figure 17 and Figure 18, Figure 19, Figure 20 and Figure 21 provide a step-by-step visualization of the cooperative manipulation strategy produced by each optimizer, revealing how the same pick-and-place objective is achieved inside the rack-like workspace.

For PSO, the motion shows a clear two-phase coordination pattern: the object is first stabilized with dominant support from the left arm, and then a reorientation is executed around the right arm (i.e., the right gripper acts as the effective pivot while the left arm adjusts/repositions). This yields a compact maneuver where rotation is concentrated in a localized region, which is consistent with the more time-efficient and refined trajectories observed previously.

In contrast, WOA follows a more “geometric” sequence: the payload is lifted almost vertically in a straight segment to increase clearance, and only afterwards is the object rotated about the right arm before placement. This strategy prioritizes safety margin (clearance-first) and produces a feasible motion but typically implies a less direct route and slower refinement compared to PSO.

Finally, GOA exhibits the most conservative coordination, with larger object excursions and a less compact reorientation/transfer through the constrained aisle. The resulting manipulation appears to rely on broader motions to maintain clearance, which qualitatively explains why GOA achieves feasibility but tends to remain less time-efficient in the final solutions.

### 6.4. Study of the Grasping Forces

Although the study is primarily kinematic, we further complement the verification of the obtained solutions with a lightweight post-processed grasp-effort analysis. Specifically, we assume a rigid cardboard box of 7.5 kg transported under an idealized lateral two-gripper grasp (no slip) and model each end effector as being covered with a thin anti-slip rubber pad. From a practical standpoint, achieving higher payloads while guaranteeing the no-slip condition strongly benefits from maximizing the available contact friction; therefore, in real deployments, it is recommended to use high-friction interface materials (e.g., by covering the end effector with an anti-slip rubber layer or textured pads) to increase μ and reduce the required clamping force for a given tangential load. Accordingly, we adopt a static friction coefficient of μ = 0.8 for rubber–cardboard contact under dry/clean conditions [26]. The analysis uses the executed box trajectory to compute the translational acceleration of the payload and the corresponding net force $F_{req}=m(a-g)$ in the world frame. At each time instant, this required force is then balanced by both grippers by solving a minimum-effort equilibrium subject to a friction-limited no-slip constraint at each contact, $∥t_{i}∥\leq \mu N_{i}$, where $N_{i}$ is the gripper’s normal (clamping) force and $t_{i}$ is the tangential component available to prevent sliding.

**Figure 22:**
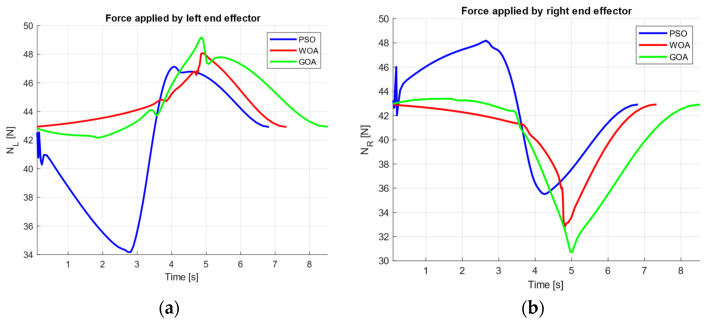
Normal forces required at the grasp during trajectory execution for the best solutions found by PSO (blue), WOA (red), and GOA (green): (**a**) left gripper normal force $N_{L}$; (**b**) right gripper normal force $N_{R}$.

Figure 22 reports the resulting normal-force profiles for the best PSO/WOA/GOA solutions: Figure 22a shows the left gripper normal force $N_{L}$, and Figure 22b shows the right gripper normal force $N_{R}$. Importantly, this post-analysis indicates that, for a 7.5 kg payload, the cooperative manipulation can be executed with feasible contact-effort levels by sharing the load between both GoFa 5 manipulators, thus exceeding the nominal single-arm payload rating (5 kg).

## 7. Conclusions

This work demonstrates that a feasibility-first, bio-inspired optimization framework can reliably generate collision-free and time-efficient cooperative motions in rack-constrained dual-arm pick-and-place tasks. Under identical bounds and stopping criteria, PSO, WOA, and GOA consistently identified feasible (collision-free) trajectories, confirming the suitability of population-based metaheuristics for constraint-dominated dual-arm bimanipulation problems.

Clear performance differences emerged in both feasibility discovery and solution refinement. PSO reached feasibility fastest (4 iterations, 19 s) and achieved the best final solution (BCV = 0.1365) with the shortest estimated execution time (TET = 6.825 s). WOA and GOA required 10 iterations to reach feasibility and converged to slower final motions (TET = 7.330 s and 8.525 s, respectively), with higher BCV values. Given the comparable iteration throughput across algorithms, these differences are attributed to their optimization dynamics—particularly their exploitation capability within the feasible region—rather than computational speed.

The results indicate that, in narrow rack-like workspaces, sustained refinement within the collision-free basin is critical for translating feasibility into time-efficient cooperative motion. Trajectory visualizations corroborate this conclusion: PSO generated more compact transfers and localized reorientations, whereas WOA and GOA favored more conservative clearance-building strategies that preserved safety at the expense of execution time.

Future work will extend validation to additional workspace layouts, payload geometries, and constraint configurations, incorporate richer performance metrics (e.g., torque/energy proxies and robustness margins), and assess real-world deployment to evaluate tracking accuracy, repeatability, and safety under sensing and actuation constraints.

## Data Availability

Data are available upon reasonable request.

## Nomenclature

Symbol	Description
PSO	Particle Swarm Optimization
WOA	Whale Optimization Algorithm
GOA	Gazelle Optimization Algorithm
$x_{i}^{t}$	Position (state) of particle/agent i at iteration t
$x_{i}^{t}$	Velocity of particle/agent i at iteration t
ω	Inertia weight (PSO)
$c_{1},c_{2}$	Cognitive and social acceleration coefficients (PSO)
$r_{1},\;r_{2}$	Random scalars (PSO), typically uniform
$p_{i}^{t}$	Personal best position of particle i up to iteration t
$g^{t}$	Global best position found by the swarm up to iteration t
A, C	Coefficient vectors in WOA (step modulation)
$r_{i}$	Random scaling coefficient in GOA update
$s_{i}$	Exploitation (decaying) coefficient in GOA update
λ	Random scaling parameter in GOA
η	Initial exploitation scaling parameter in GOA
α	Exponential decay rate in GOA
δ	Mutation/perturbation amplitude in GOA
x, y, z	Cartesian position components of box reference frame
roll, pitch, yaw	Euler angles describing box orientation
$p_{L},$ $p_{R}$	Left and right grasp frames/poses induced by box pose
$n_{\mathrm{vars}}$	Total number of optimization decision variables
$n_{\mathrm{total}}$	Total number of waypoints in the box trajectory
m	Number of fixed waypoints (task constraints, e.g., start/end)
n	Number of optimized waypoints
{W}	World reference frame
$W_{T_{0}}$	Homogeneous transform of objects in world frame
$W_{R_{o}}$	Object rotation matrix in world frame
$W_{p_{o}}$	Object position in world frame
$\;R_{e_{i}}^{o}$	Relative grasp orientation for arm i
$\;T_{e_{i}}^{o}$	Constant grasp transform from object frame to end-effector frame
$W_{i}$	Admissible workspace set of end-effector positions for arm i
$p_{box}$	Box position
$q_{R},\;q_{L}$	Joint configurations of left/right arms
$p_{L},\;p_{R}$	Left/right end-effector target positions induced by box pose
d	Half lateral separation distance from box center to each grasp point
$f_{c}$	Cost function
$\omega_{O}$	Set of obstacles in workspace
$O_{j}$	Obstacle j (convex body/convex component)
$N_{O}$	Number of obstacles
GJK	Gilbert–Johnson–Keerthi collision detection method
A, B	Convex sets used in collision test
A ⊖ B	Minkowski difference of sets A and B
Ø	Empty set
IFFS	Iterations to First Feasible Solution
TFFS	Time to First Feasible Solution
BCV	Best Cost Value (best achieved fitness)
TET	Trajectory Execution Time (of feasible solution)
IT	Iteration Throughput (iterations per hour)

## Appendix A

PSO, WOA, and GOA were configured using standard hyperparameter settings recommended in the seminal references and commonly adopted for continuous optimization problems with comparable dimensionality [27,28,29,30,31]. In this study, each adjustable waypoint $p^{box}\in SE(3)$ is defined by six decision variables $[x,y,z,roll,pitch,yaw{]}^{⊤}$. The total number of optimization variables is therefore $n_{vars}=6\;(n_{total}-m),$ as given in Equation (12). In our case, the trajectory contains $n_{total}=5$ waypoints, with m=2 fixed poses (pick and place), so $n_{vars}=6\;(5-2)=18$ decision variables. The hyperparameters reported in Table A1 were selected to match these standard recommendations for low-to-moderate dimensional continuous spaces and were kept fixed across runs to preserve fairness and reproducibility.

**Table A1:** Hyperparameter settings used for PSO/WOA/GOA in this study.

Algorithm	Hyper Parameter	Meaning	Value
PSO	ω	Inertia weight (Equation (2))	[0.1,1.1]
	$c_{1}$	cognitive coefficient (Equation (2))	1.49
	$c_{2}$	Social coefficient (Equation (2))	1.49
	$r_{1},\;r_{2}$	Random scalars (Equation (2))	U(0,1)
WOA	a	exploration–exploitation control (drives A)	a(t)=2−2t/1000 (linear decay 2→0)
	A	Coefficient in Equation (5)	$A=2ar_{1}-a$
	C	Coefficient in Equation (5)	${C=2r}_{2}$
	p	switch between encircling/spiral	U(0,1)
	b	spiral shape in Equation (6)	1
	l	spiral random parameter in Equation (6)	$l=\left(a_{2}-1\right)rand\left(0,1\right)+1$
GOA	λ	random scaling in Equation (8)	1
	η	scale in Equation (9)	1
	α	decay rate in Equation (9)	1
	δ	mutation amplitude in Equation (11)	1

Furthermore, the common experimental settings are the following: population size N=50 agents, maximum iterations $I_{max}=1000$.
